# Aneurysmal Bone Cyst of the Proximal Femur and Its Management - A Case Report

**DOI:** 10.7759/cureus.991

**Published:** 2017-01-23

**Authors:** Chirag Kapoor, Malkesh Shah, Rishit Soni, Jagdish Patwa, Aditya Merh, Paresh Golwala

**Affiliations:** 1 Orthopaedics, Sumandeep Vidyapeeth, Vadodara, Gujarat

**Keywords:** cyst, sclerosing agent, ender's nail, dhs, dynamic hip screw, aneurysmal bone cyst

## Abstract

Aneurysmal bone cyst (ABC) is a benign, expansile, non-neoplastic lesion of the bone, characterized by channels of blood and spaces that are separated by fibrous septae. Giant ABC is an uncommon condition and can be difficult to handle because of the destructive effect of the cyst on the bones and the compressive effect on the nearby structures, especially in weight-bearing bones of the body. We report a case of a giant aneurysmal bone cyst in the proximal femur of a six-year-old child, which was treated with a sclerosing agent and ender's nail fixation first. There was recurrence after 13 months. It was then curetted out extensively, the cavity was filled with bone graft, and fixation with a dynamic hip screw (DHS) was done. At 19 months follow-up, the lesion had subsided and patient was walking pain-free without any deformity. We suggest this method of treatment to be worthwhile for ABC at this site and at this age.

## Introduction

Aneurysmal bone cyst (ABC) is a benign, expansile, non-neoplastic lesion of the bone, characterized by channels of blood and spaces that are separated by fibrous septae. ABC is a benign lesion, but malignant transformation has been reported in some cases [1]. Seventy-five percent of the lesions occur in the first two decades of life, and almost 95% occur in the first three decades [2]. Giant ABC is an uncommon condition and can be difficult to handle because of the destructive effect of the cyst on the bones and compressive effect on the nearby structures, especially in the weight-bearing bones of the body.

Several treatment modalities are described for ABC, such as curettage, curettage with cementation or bone grafting, fibrosing agents or bone marrow injections, arterial embolization, adjuvant cryotherapy or radiotherapy, demineralized bone matrix applications, and segmental or en bloc resections. En bloc resection has the advantage of the lowest recurrence rate, which is as low as 0% [3].

We report a case of a giant aneurysmal bone cyst in the proximal femur of a young child, which was treated with two different modalities because of recurrence.

## Case presentation

A six-year-old female patient presented with pain in the right hip of four months duration, along with difficulty in walking. There was no other significant contributing history. On local examination, there was tenderness on deep palpation of the right hip and restricted range of hip movements. The overlying skin was normal with no redness or dilated veins.

**Figure 1:**
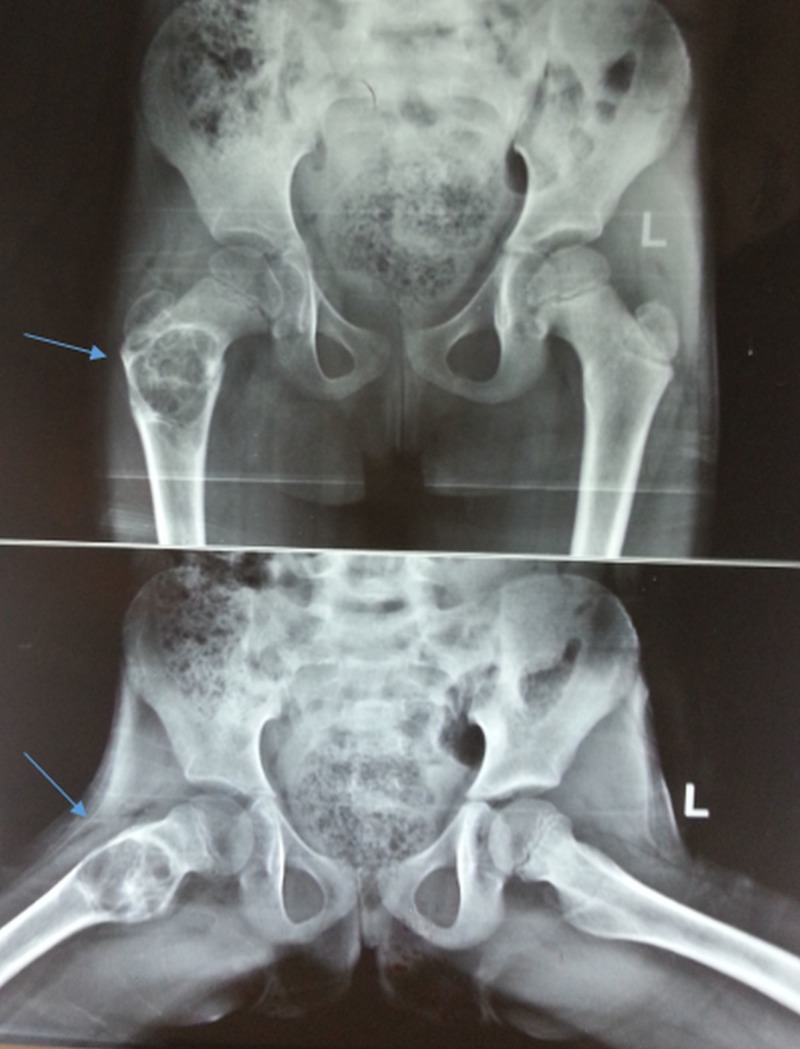
Preoperative x-ray (anteroposterior and lateral) Plain radiographs show lytic lesion in proximal portion of the right femur

Plain radiographs revealed a well-defined, expansile, lytic lesion involving the proximal portion of the right femur in the trochanteric and subtrochanteric region approximately 5 cm x 5 cm in size (Figure 1).

**Figure 2:**
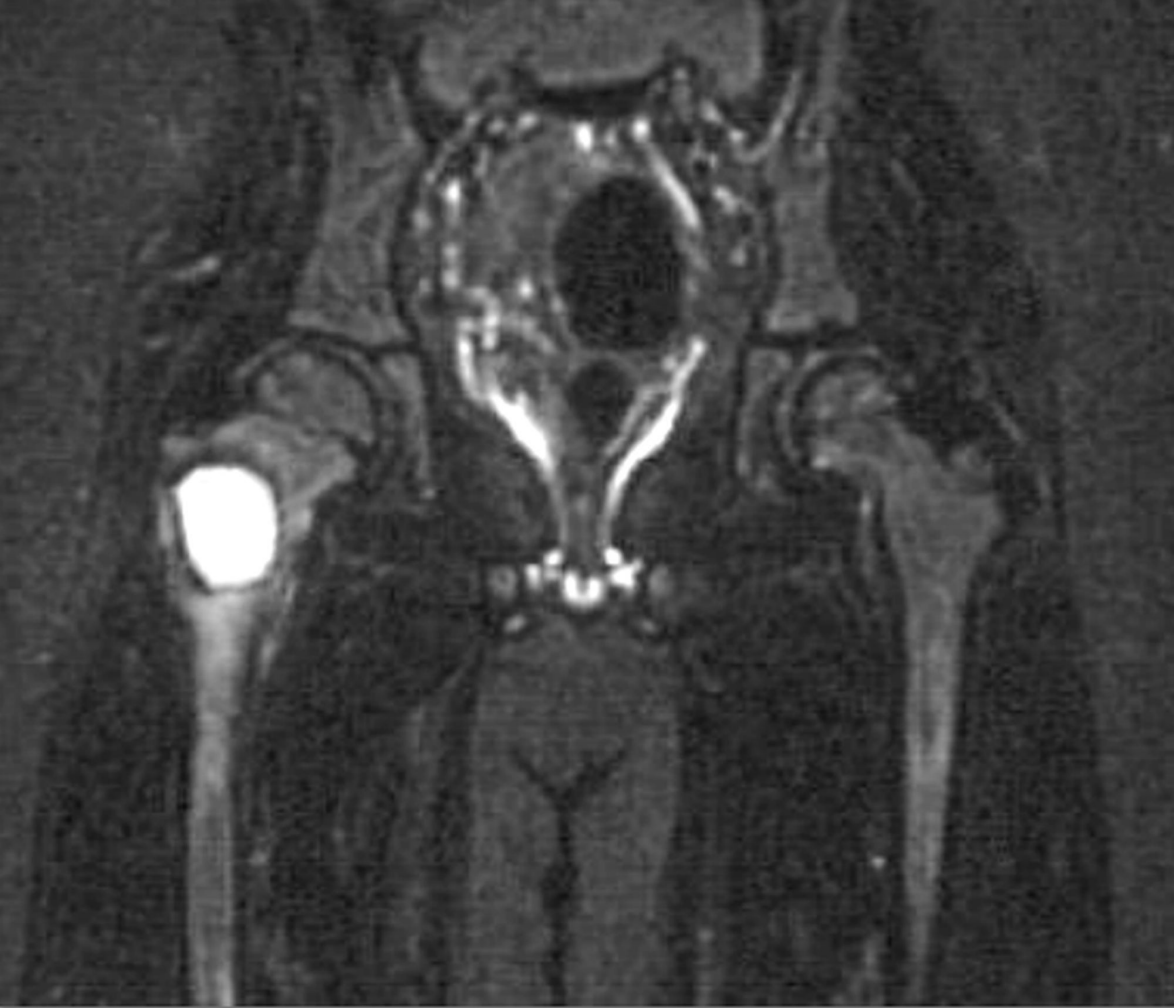
MRI T2-weighted section Shows a lytic lesion in the proximal portion of the right femur.

An MRI of both hips was done, which showed a hyperintense lesion in the proximal end of the shaft of the right femur with internal septations on a T2-weighted image. It appeared hypointense on the T1-weighted image and showed inhomogenous enhancement on the contrast study, which was suggestive of an aneurysmal bone cyst (Figure 2).

**Figure 3:**
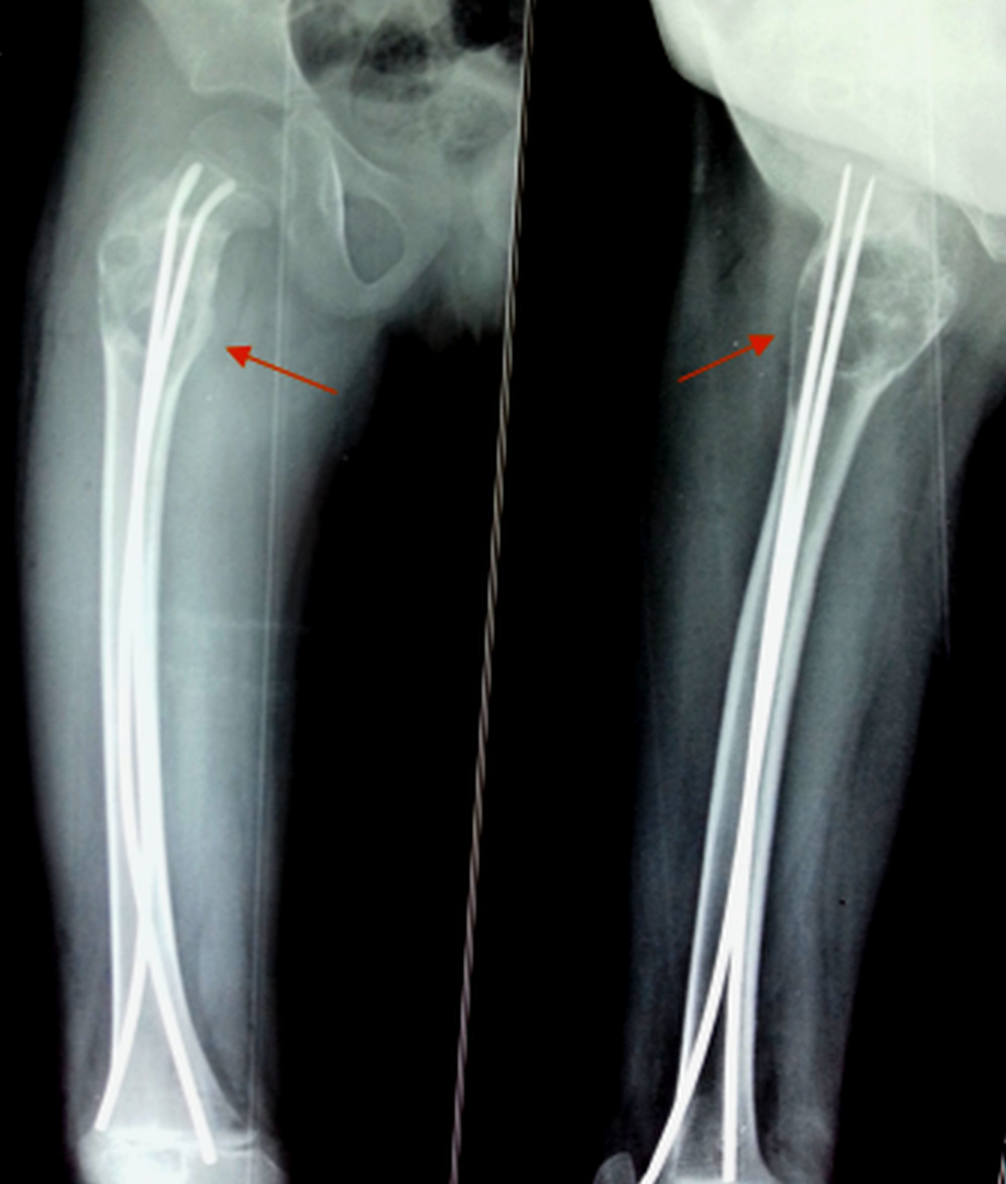
Postoperative radiograph (anteroposterior and lateral views) Shows fixation with ender’s nails after injecting a sclerosing agent in the lesion.

**Figure 4:**
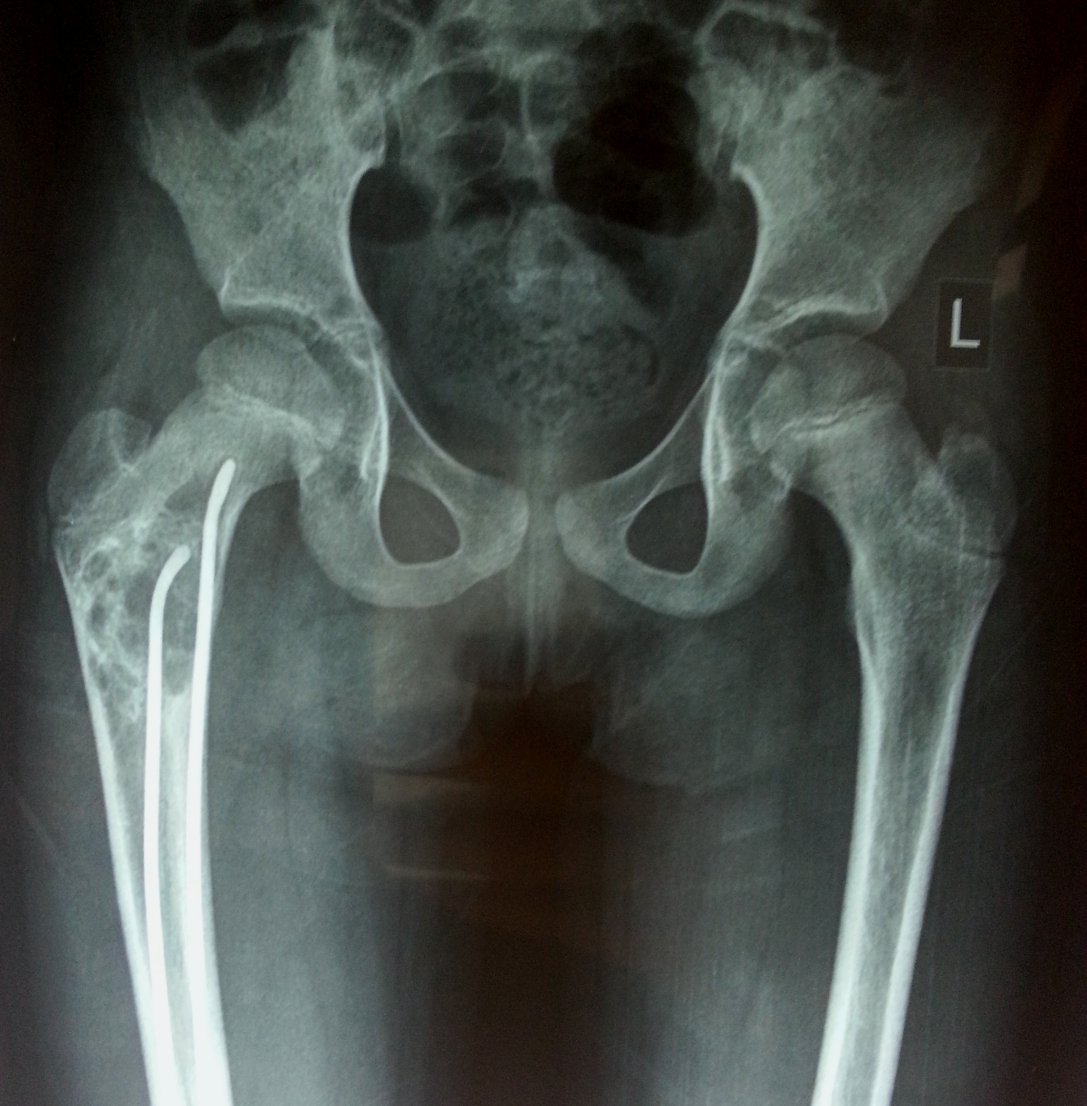
Follow-up radiograph at 13 months Shows recurrence of the lesion with ender’s nails in situ.

The patient was prepared for surgery after obtaining a written informed consent from the parent. We aspirated the lesion under the guidance of an image-intensifier and then injected polidocanol, a sclerosing agent, percutaneously. This was augmented by fixation with two ender’s nails for prophylactic stabilization of the affected region (Figure 3). It was sent for histopathological examination, which confirmed it to be an aneurysmal bone cyst. After 13 months, the patient again had the same complaints. Radiographs were repeated, which showed a recurrence of the lesion (Figure 4).

**Figure 5:**
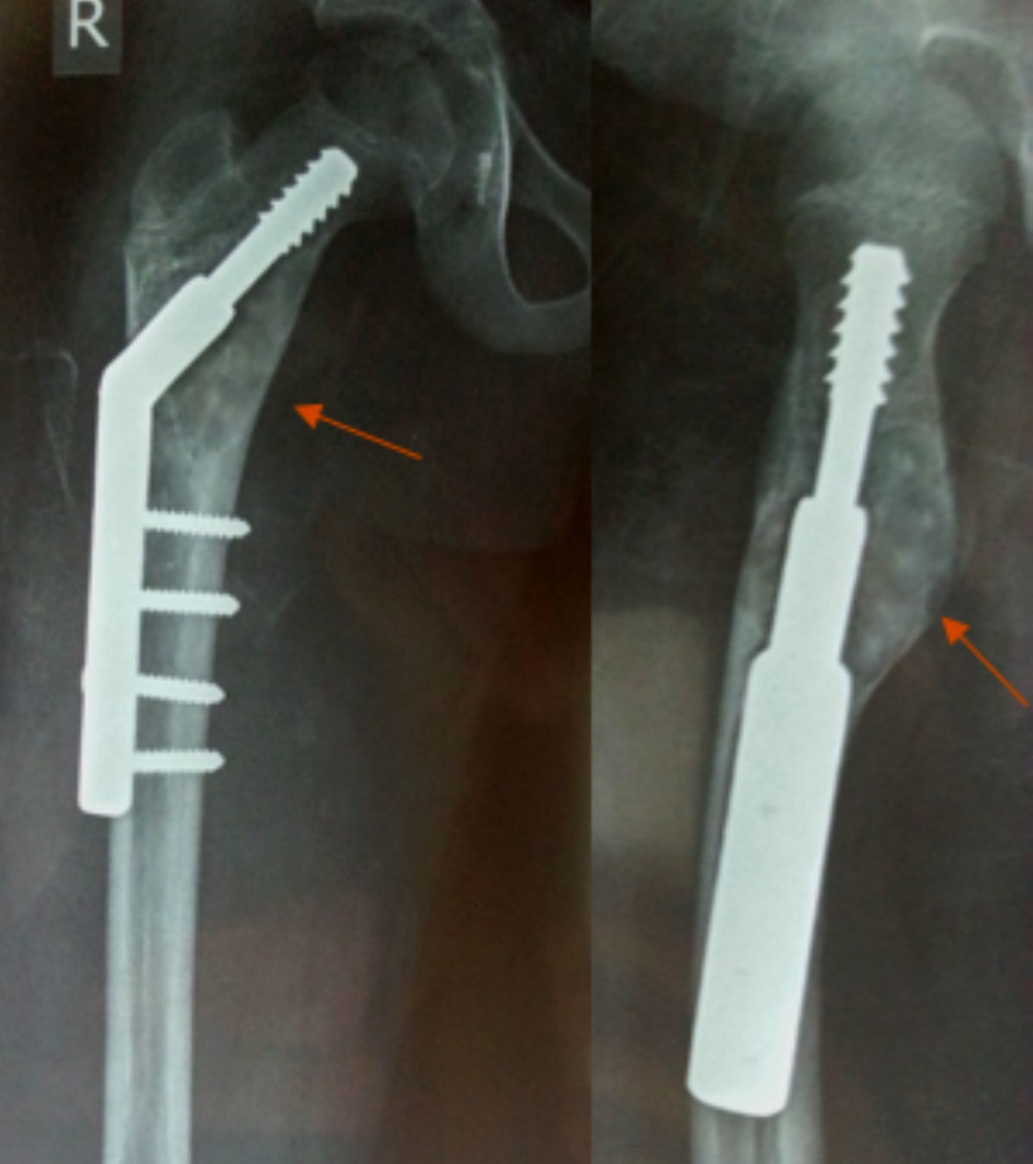
Postoperative radiograph (anteroposterior and lateral views) Shows curettage of the lesion with bone grafting and fixation with dynamic hip screw (DHS).

Informed patient consent was obtained for surgery from the parent. The ender’s nails were removed, an extensive curettage of the tumour was done, and the bone defect was filled with an autogenous cancellous bone graft, along with prophylactic fixation with a dynamic hip screw (DHS) (Figure 5).

She was kept non-weight-bearing for six weeks and then using a mobilized non-weight-bearing with a walker after that until the bone graft was seen to be incorporated radiologically. After 12 weeks, x-rays showed healing of the lesion and incorporation of bone graft, after which patient was allowed full weight-bearing.

**Figure 6:**
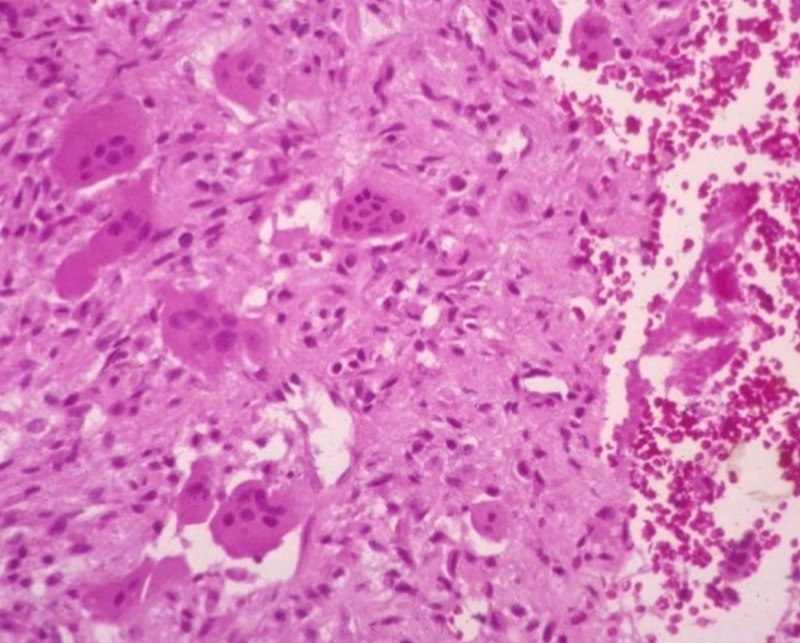
Histopathology slide Showing stroma consisting of proliferative fibroblasts, spindle cells, areas of osteoid formation, and uneven large cystic spaces.

The histopathological features showed stroma consisting of proliferative fibroblasts, spindle cells, areas of osteoid formation, and uneven, large cystic spaces filled with blood and separated by fibrous septa alternating with solid areas (Figure 6).

**Figure 7:**
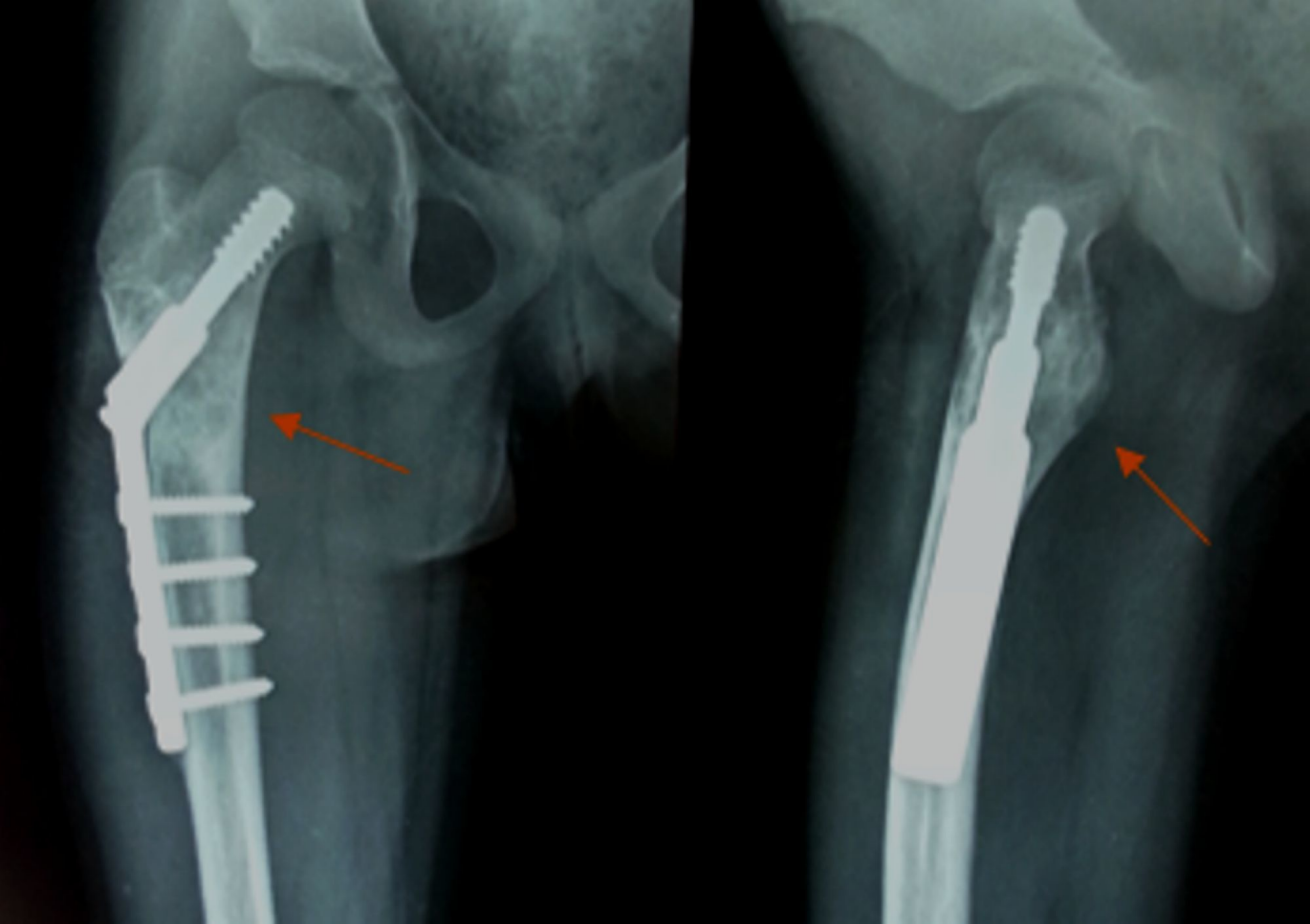
Final follow-up radiographs at 19 months Shows complete healing of the lesion and incorporation of the bone graft.

At 19 months follow-up, the patient had no pain and was walking without support with a full range of hip movements. No signs of recurrence were seen radiologically (Figure 7).

## Discussion

Aneurysmal bone cysts can occur in any bone, but it is more commonly located in the metaphysis of long bones, especially weight-bearing ones. It can present as a primary or secondary lesion (e.g., associated with chondroblastoma or osteoblastoma). Primary ABC’s arise de novo. Although ABCs are typically located in the metaphysis, because of the aggressive nature of this tumour, physeal involvement or extension may occur, resulting in growth plate disturbances and subsequent development of deformities [4]. Radiographically, an ABC is a lytic and expansile lesion that presents with cortical thinning and septations and shows fluid-fluid levels on MRI, which was also seen in our patient [4].

The optimal treatment for ABC’s is debatable. In spite of the number of techniques reported in the literature, there remains a recurrence rate that ranges from 5% to greater than 40% [5]. Sclerosing substances and bone substitutes are less effective than conventional curettage [6]. At present, curettage and filling the cavity with bone graft or polymethylmethacrylate is the principal modality used [6].

Large defects after resection of giant aneurysmal bone cysts are difficult to treat. Many reconstructive options are available to fill these defects and to provide structural integrity to the bone, such as allogenic or autogenic bone grafts and many different bone substitutes [7]. The incorporating process of allograft is slower and less complete than that with autografts due to a low-grade immune response or a lack of osteocytes in the graft or both [8]. Vascularized bone grafts have been suggested as the best method to replace large bone defects due to their ability for faster incorporation and remodeling, but it is a technically demanding procedure [9]. Our choice was to use non-vascularized autogenous cancellous bone grafts as they are technically easier to harvest and provide excellent structural bone support. Also, successful long-term results of surgical en-bloc resection and replacement with nonvascularized, autologous bone graft have been reported in the literature [10]. In the present case, the final construct obtained was stable and allowed progressive weight-bearing without graft failure.

## Conclusions

Treatment for aneurysmal bone cysts should be individualized, taking into account the location of the tumour, its aggressiveness, and its extent. We recommend that aggressive and large aneurysmal bone cysts in proximity to the physis should be extensively curetted and filled with autogenous bone graft and prophylactic fixation should be done to prevent pathological fracture and to give support to the graft.
